# The Relationship between Visual Field Global Indices and Retinal Nerve Fiber Layer Thickness in Healthy Myopes

**DOI:** 10.1155/2014/431901

**Published:** 2014-11-10

**Authors:** Yuan-zhi Yuan, Chen-li Feng, Bao-yue Li, Min-qian Shen, Xiu-ping Chen, Chen-hao Zhang, Chun-qiong Dong, Fei Yuan

**Affiliations:** ^1^Department of Ophthalmology, Zhongshan Hospital, Fudan University, Shanghai 200032, China; ^2^Centre for Evidence-Based Medicine, Fudan University, Shanghai 200032, China; ^3^Department of Biostatistics, Erasmus University Rotterdam, 3015 GE Rotterdam, The Netherlands

## Abstract

The aim of the current study was to investigate the association between the thickness of the retinal nerve fiber layer (RNFL) and central visual field indices in otherwise healthy myopes. In total, 57 otherwise healthy subjects were cross-sectionally studied. General ophthalmic examinations, refractive measurements, RNFL thickness by spectral domain optical coherence tomography (OCT), and central visual fields were examined. Linear models were used to assess the associations. In this young and mid-aged population, the mean spherical equivalent was −4.79 (SD 1.66) and −4.59 (SD 1.88) diopters in the right and left eyes, respectively. Approximately 7% to 14% of the eyes showed the average RNFL thickness out of the normal range. The temporal RNFL was remarkably thicker, whereas the nasal RNFL was thinner. The higher the refractive error, the thinner the RNFL thickness. A thicker overall RNFL was significantly associated with decreased mean sensitivity and increased mean defect, and further adjustments for age, sex, refractive error, optic disk area, or ocular magnification did not change the association. Although nonpathologic myopia does not significantly affect central visual field global indices, its effects on the RNFL may be linked with performance on the central visual field test.

## 1. Introduction 

It has been well documented that a high prevalence of myopia exists in East Asia and the western Pacific regions [1–5], and studies have indicated that myopia is an independent risk factor for primary open angle glaucoma (POAG) [6–8]. However, making a glaucoma diagnosis can sometimes be particularly difficult in myopes. Optic disc changes due to myopia (e.g., tilt or rotation of the optic disc and/or peripapillary chorioretinal atrophy) make the discrimination of a glaucomatous optic nerve head more difficult. In addition, myopia has been reported to be associated with visual field impairment [9].

Recently, retinal never fiber layer (RNFL) thickness has become more widely used for the diagnosis and follow-up of glaucoma. However, studies have consistently shown that myopia affects the RNFL thickness [10–14], especially in cases assessed by optical coherence tomography (OCT) [15, 16]. Myopia also exerts an impact on the RNFL distribution [11, 12]. Quantifying the relationship between RNFL and visual function in myopia would be important in understanding the sequelae of myopia and in aiding glaucoma diagnosis in myopes. However, such structure-function association studies are seldom performed in nonpathologic myopia.

In the current study, we investigate the association between RNFL thickness and visual field indices in a group of otherwise healthy myopic persons.

## 2. Participants and Methods

### 2.1. Study Population

The current study had a cross-sectional design of otherwise healthy nonpathologic myopic patients. The participants were recruited by advertisement in Zhongshan Hospital. Briefly, the inclusion criteria included the following: (1) male or female aged 18 to 55 years old, (2) mild to high myopia with spherical equivalent ≥−10.00 D, (3) best corrected visual acuities ≥20/20 in both eyes, (4) otherwise healthy, and (5) without a history of ocular trauma or surgery. We excluded those who (1) showed any signs in the fundus consistent with pathologic myopia, such as choroidal neovascularization, macular hemorrhage, Fuchs spot, lacquer cracks, disciform degeneration, or chorioretinal atrophy or (2) required the use of ophthalmic drug treatment. The study was approved by the Ethics Committee of Zhongshan Hospital, Fudan University, and was conducted according to the tenets of the Declaration of Helsinki. Written informed consent was obtained from all participants. The study was conducted in Zhongshan Hospital affiliated with Fudan University, Shanghai, China, from October 2010 to March 2011.

### 2.2. General Ophthalmic and Refractive Examinations

General ophthalmic examinations were performed by a trained ophthalmologist in the outpatient clinic of Zhongshan hospital, Fudan University. Intraocular pressure was measured by a noncontact pneumotonometer (TX-F, Canon, Tokyo, Japan). Corneal curvature, anterior chamber depth, and axial length were measured without contact according to standard operating procedures (IOLMaster 1322-734, Carl Zeiss, Jena, Germany). Patients' refractive corrections were determined by both objective and subjective refractions (Feng CL, Zhang CH).

### 2.3. Fundus Photography, OCT, and Perimetry

The general ophthalmic and refractive examinations were followed by fundus photography, OCT, and perimetry, sequentially. The fundus was photographed centered at the optic disc in each participant by a nonmydriatic fundus camera (TRC-NW100, Topcon, Tokyo, Japan). Then, the area and maximum and minimum diameters of the optic disc were delineated and measured manually (Yuan YZ) via IMAGEnet Professional (Topcon, Tokyo, Japan).

A spectral-domain HD-OCT (Ver. 4.5, Cirrus HD-OCT 4000, Dublin, CA, US) was used in the current study. Each eye of every participant had its optic disc scanned in a random sequence following standard imaging procedures. Specifically, imaging was obtained using a cube scan featuring 200 × 200 axial horizontal scans (pixels) centered on the optic nerve. The image quality was assessed by the examiner immediately after each scan. Only well-focused, well-centered images without any eye movement and with a signal strength of 7/10 or greater were used. The RNFL parameters of the printout retained for analysis were of inferior, superior, nasal, temporal, and average thickness.

Central visual field tests using the dG1X program and a dynamic strategy were performed in a dark exam room using an automated perimeter (Octopus 1-2-3; Interzeag, Schlieren, Switzerland). To perform the exam, each participant was asked to wear his/her glasses or contact lenses, whichever he/she had; if needed, trial lenses were provided. Before the test, the corrected visual acuity, glasses and contact lenses (when applicable) of each participant were examined by the optometrist (Zhang CH) for suitability. If the corrected visual acuity was under 20/20 and/or any problem was noted in the glasses or contact lenses, best corrected trial lenses were used. The mean defect (MD), mean sensitivity (MS), and loss of variance (LV) were acquired from the readouts. The reliability factor (RF) was used for quality control; for any test with an RF exceeding 20%, retest was required.

### 2.4. Statistical Analysis

Statistical analyses were based on data from both of the participant's eyes. The refraction was expressed by spherical equivalents (SE), which were the sphere plus 1/2 the cylinder. High myopia was defined as SE exceeding −6.00 diopters. The average central corneal curvature was expressed by the mean of the 2 principle meridians. The normally distributed continuous variables were expressed as the means and standard deviations (SDs), whereas medians and interquartile range were used to describe the nonnormal data. Visual field indices were treated as continuous variables, and the RNFL thickness was treated both as a continuous variable and as a categorical variable (below 1%, 1–5%, 5–95% and above 95% in the distribution of normal, based on the build-in normative data of OCT manufacturer).

Type C intraclass correlation coefficients (ICC) were employed to describe the correlation and consistency between right eyes and left eyes using two-way mixed models. The linear mixed regression model was used to account for the intraindividual binocular correlation. The associations between the RNFL thickness and visual field indices were first analyzed separately for the right and left eyes by general linear models and later by combining the data and examining them with linear mixed models. A scaling factor based on Bennett's formula [17, 18] was included in one model to adjust for ocular magnification. Bennett's formula relies on the axial length to correct for ocular magnification. The ocular magnification is estimated based on the location of the second principal point and its normal spatial relationship to the nodal point. Given the default axial length (AL, 24.46 mm) and refraction (0 D) for a magnification of 1 with the SD-OCT system, an individual scaling factor can be expressed as (24.46 − 1.82)/(*AL*⁡−1.82).

The Pearson correlation between RNFL thickness and MS was used to calculate the sample size [19]. A sample size of 58 was able to achieve 81% power to detect a difference of −0.36 between the null hypothesis correlation of 0 and the alternative hypothesis correlation of 0.36, using a two-sided hypothesis test with a significance level of 0.05. All statistical analyses were completed by SPSS (Windows ver. 15.0; SPSS Inc., Chicago, IL, USA).

## 3. Results

### 3.1. Demographic and Biometric Characteristics

In total, sixty eligible participants were recruited into the trial. One participant declined the OCT exam, and 2 more refused perimetry. Finally, 57 participants (52 females and 5 males) were included in our data analysis.

The mean age of the participants was 28.3 (ranged 19–46, SD 5.8) years. The mean SE was −4.79 (ranged −1.80 to −8.80, SD 1.66) diopters in the right eyes and −4.59 (ranged −0.50 to −9.00, SD 1.88) diopters in the left eyes. High myopia was noted in 23.2% of the eyes.

The RNFL thickness, visual field indices, and other ocular biometric measurements are presented in Table 1.

### 3.2. Binocular Correlations

Both the RNFL thickness and visual field indices were highly correlated intraindividually. The ICC for the average RNFL thickness was 0.89 (95%CI 0.81–0.94). For MD and MS, the corresponding ICCs were identical, at 0.91 (95%CI 0.85–0.95).

### 3.3. RNFL and Refractive Errors

**Figure 1:**
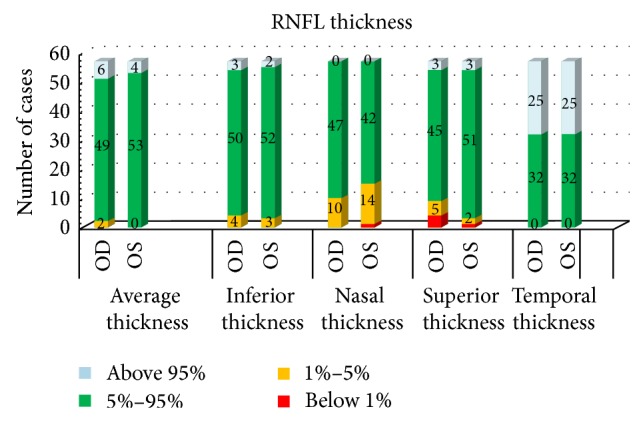
The numbers of cases in 4 categories of retinal nerve fiber layer (RNFL) thickness (below 1%, 1–5%, 5–95% and above 95% in the distribution of normal based on Cirrus build-in RNFL Normative Database), overall average and quadrant specific RNFL thicknesses. (Left) Although most eyes fell into the normal ranges, over 40% of eyes had thick temporal RNFL (Right), and approximately 20% of the eyes had thin nasal RNFL (Middle). OD:* oculus dexter* (the right eye); OS:* oculus sinister* (the left eye).

Comparing to the normative database, the overall and quadrant RNFL thicknesses of each participant fell into 4 categories: below 1%, 1–5%, 5–95% and above 95% in the distribution of the reference values. The categories of RNFL thickness, both overall and quadrant-specific, are shown in Figure 1. For the average RNFL thickness, 86.0% of the right eyes and 93.0% of the left eyes were categorized into the normal (5–95%) group. The temporal RNFLs were significantly thicker (43.9% of the eyes were above 95% normative distribution), whereas the nasal RNFLs were much thinner (17.5% of the right eyes and 26.3% of the left eyes were classified as below 5% normative distribution).

As revealed by the linear mixed model, the higher the refractive error, the thinner the RNFL thickness. A one-diopter increase in the refractive error was associated with 1.61 (95%CI 0.72–2.50) *μ*m thinning of the RNFL. The association between optic axis length and RNFL was also statistically significant; a 1-mm increase in axis length was accompanied by 3.33 (95%CI 1.44–5.22) *μ*m thinning of the RNFL.

### 3.4. Myopic and Visual Field Indices

Neither clinically nor statistically significant associations were found between the myopia and global visual field indices (Table 2). A 1-diopter change in the refractive error was associated with only a 0.02 (95%CI −0.14, 0.18) dB change in the MS and a 0.01 (95%CI −0.16, 0.15) dB change in the MD. The corresponding values are 0.03 (95%CI −0.30, 0.37) dB and −0.06 (95%CI −0.39, 0.27) dB for MS and MD, respectively, per 1 mm increase in axial length.

### 3.5. RNFL Thickness and Visual Field Indices

**Figure 2:**
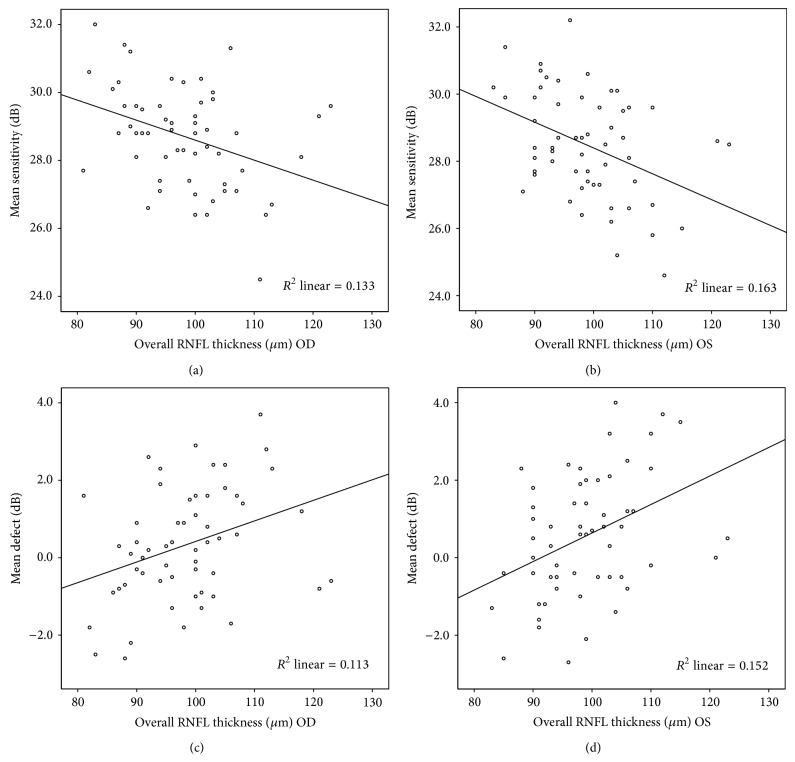
Scatter plots of overall retinal nerve fiber layer (RNFL) thickness and visual field indices (mean sensitivity (MS) and mean defect (MD)). (Top) The MS decreased with the increase of the overall RNFL thickness in right eyes (a) and left eyes (b), while the MD showed an inverse correlation with the RNFL thickness in both eyes ((c) and (d)). OD:* oculus dexter* (the right eye); OS:* oculus sinister* (the left eye).

A significant association was observed between the overall RNFL thickness and global visual field indices. A thicker overall RNFL was significantly associated with a decreased MS and increased MD. Adjusting for age, sex, refractive error, optic disc area, or ocular scaling factor had no effect on this association (Figure 2, Tables 3 and 4).

## 4. Discussion

Myopia is an independent risk factor for open angle glaucoma [6–8]. However, the morphologic and functional changes of the optic nerve and RNFL in myopic patients can complicate the diagnosis of glaucoma. It is therefore imperative to understand the RNFL parameters and their potential impact on visual field indices in normal myopic patients.

We show in our current study a negative correlation between refractive errors and the RNFL thickness. Although the overall thickness of RNFL mainly fell (86%~93%) within the normal range, the nasal thickness tended to be thinner, and the temporal region appeared thicker (Figure 1), potentially indicating a temporal rotation of RNFL in myopic eyes. Our data also showed that the average RNFL thickness was decreasing with the optic axis length or myopic refractive error. Similar findings were also reported. Leung et al. [13], Rauscher et al. [12], Kim et al. [10], Kang et al. [11], and Wang et al. [15] found that the longer axial length or the higher refractive error, the thinner the RNFL; a significantly thicker RNFL in the temporal quadrant in myopes has also been reported [10, 11, 13]. The coherence among studies suggests a characteristic pattern of RNFL in myopic eyes. The elongation of the globe due to myopia leads to the retinal nerve fibers stretching and thinning; the nasally located optic disc moves laterally in this process. We postulate that the nerve fibers in the nasal quadrant stretch either superiorly or inferiorly, whereas the superior temporal and inferior temporal nerve fibers move centrally toward the horizontal meridian or macula in a nonlinear fashion. During this process, the nasal quadrant loses nerve fibers, and the temporal quadrant gains, finally causing the RNFL to tend to be thinner nasally and thicker temporally. The superior and inferior regions gained and lost fibers simultaneously; therefore, the thicknesses vary depending on the balance between gaining and losing (Figure 1).

The association between myopia and global visual field indices has been debated in the literature. Martin-Boglind [20] observed that the mean resolution threshold significantly correlated with the degree of myopia in the central 30-degree field. Huang [21] showed that a group of nonpathologic high myopic patients had refractive degrees and axial lengths that were significantly positively correlated with total visual field loss. In Araie et al.'s study of glaucoma patients, however, myopic power affected the mean deviation either way [22]. In one of his papers, Rudnicka and Edgar [23] stated that a sensitivity decline of the central field occurred in subjects with axial lengths > 26 mm and >5 D of myopia. Aung et al. [24] found that the prevalence of visual field defects was surprisingly low in young males with myopia. Czepita and Chmielewska [25] reported a positive association between refractive error and visual field defects in low and medium myopia. A group of glaucomatous suspects of Chinese ancestry who had not presented for a check of their glaucomatous progression for up to 7 years showed no correlation between axial length and mean deviation on visual field testing [26]. In the current study, no association between the refractive error and global visual field indices was revealed. This discrepancy among studies may be due to multiple factors. The subjects' ages, the degree of concomitant myopia, and the controls varied from study to study. Different perimetries [27] and refractive corrections [24, 28] were also reported to have influenced the association between refractive error and visual field indices.

There are few studies investigating the association between the retinal nerve fiber layer and visual field in nonglaucomatous populations. Ajtony et al. [29] plotted a linear regression line of MS or MD against RNFL thickness, demonstrating a negligible degree of determination in normal (*R* ^2^ = 0.0378 and 0.0121, resp.) and preperimetric glaucoma groups (*R* ^2^ = 0.0215 and 0.0151, resp.). Taliantzis et al. [19] reported weak associations of the thickness of RNFL with visual field indices in ocular hypertension (Pearson correlation *R* = 0.303, *P* < 0.05 for MS, and *R* = −0.239, *P* < 0.1 for MD) and with preperimetric glaucoma groups (*R* = 0.323, *P* < 0.05 for MS, and *R* = −0.209, *P* > 0.1 for MD). Such mild correlations were also reported in normal and ocular hypertension groups [30]. Compared with the findings of Taliantzis and Ajtony, the current study showed (Tables 3 and 4) slightly higher correlations between RNFL thickness and visual field indices (indicated by *R*) and greater variation in the visual field indices, which can be explained by the thickness of the RNFL (indicated by *R* ^2^). In contrast to previous studies and common sense, the most interesting finding of the current study is the inverse association between RNFL thickness and visual field indices; that is, the thinner the RNFL, the better the performance on the perimetry test (higher MS and lower MD). This inverse association may partly be due to the increased attention caused by myopia. A study [31] on the correlation between myopia and visuospatial attention found that more severe myopia was associated with a better ability to quickly narrow the focus of visual attention to a small region of space. In our study, the negative association between RNFL thickness and the performance of the visual field test persisted even after adjusting the refractive power, implying that the stretching and thinning of RNFL* per se* may also affect the visual field test. Another explanation of this discrepancy includes gender [32] and race differences [33, 34]. Further large and well-controlled studies are warranted to answer this question.

On the other hand, ocular magnification can change the actual location of the measurement circle on the peripapillary retina, thereby affecting the average RNFL thickness measurements [11]. However, Nowroozizadeh et al. used customized measurement circles in normal and glaucomatous eyes and found that a correction with ocular magnification did not improve global or regional structure-function relationships [18]. Compared to Nowroozizadeh et al.'s study, the myopic subjects in the current study had longer axial length. According to Bennett's formula, axial length affects the magnification of the ocular optic system by a scaling factor expressed as (24.46 − 1.82)/(*AL*⁡−1.82) [17, 18]. Axial length is also linearly correlated with refraction. Although no individualized measurement circle was employed in the current study to correct the ocular magnification of each eye, we constructed two models that adjusted for this consideration. Beside age and sex, refraction and the area of the optic disc were adjusted in one model, and the magnification scaling factor based on Bennett's formula was added in the other model. The association between RNFL thickness and the visual field global indices did not change in either model.

Given the pattern of RNFL redistribution in myopes indicated by the current and previous studies, a sector-by-sector and point-by-point structure-function correlation study will facilitate a better understanding of the physiopathological changes in the myopic eye.

The strengths of the current study included its characterization of RNFL and its association with visual function, which were examined in a group of young and mid-aged myopes. None of the patients had comorbidities (especially the chorioretinal atrophy), ocular hypertension, or glaucoma, which allowed us to better investigate the relationship between structure and function in myopia.

There are several limitations to the current study. First, the sample size of the current study was relatively small, and there was little power for undergoing subgroup analysis. Second, as it is limited by its cross-sectional design, the current study cannot address questions on the temporal relationships of myopia versus changes in the RNFL or of RNFL thickness versus visual field test performance. According to the present knowledge, however, there is little biological plausibility favoring alternative pathways (the changes of RNFL leading to myopia, or the performance in visual field test causing the changed RNFL). Finally, as the trial lenses were not compulsory during the visual field tests for practical reasons (especially for those participants with astigmatism), the type of refractive correction could add some variation, constituting a potential confounding source. Given the relatively small sample size and the possibility for multiple interactions, the type of refractive correction could not be adjusted in the current study. However, we attempted to carefully reduce the variation caused by defocusing and inappropriate glasses/contact lenses.

In conclusion, we found in this relatively young myopic Chinese population that the RNFL was thinner in the nasal quadrant and thicker in the temporal quadrant. Further, the average RNFL thickness was independently inversely associated with visual function, as measured by visual field indices.

## Figures and Tables

**Table 1:** Retinal nerve fiber layer (RNFL) thickness, visual field indices, and other biometric parameters in the participants.

	OD	OS
Spherical equivalents (diopters)	−4.79 (1.66)	−4.59 (1.88)
High myopia (%)	23.2%	23.2%
Average central corneal curvature (diopters)	43.65 (1.34)	43.60 (1.40)
Axial length (mm)	25.39 (0.88)	25.28 (0.95)
Overall RNFL thickness (*μ*m)	98.33 (9.19)	98.96 (8.43)
Superior RNFL thickness (*μ*m)	118.61 (17.11)	121.96 (18.96)
Inferior RNFL thickness (*μ*m)	126.96 (15.92)	124.18 (15.50)
Nasal RNFL thickness (*μ*m)	62.54 (7.70)	60.88 (8.23)
Temporal RNFL thickness (*μ*m)	85.42 (15.27)	84.68 (14.74)
Mean sensitivity (dB)	28.70 (1.48)	28.48 (1.60)
Mean defect (dB)	0.33 (1.45)	0.56 (1.59)
Loss variance (dB^2^)^a^	3.60 (2.90–5.15)	3.70 (2.70–6.10)
Intraocular pressure (mmHg)	15.34 (3.02)	15.01 (3.06)

OD: oculus dexter (the right eye).

OS: oculus sinister (the left eye).

Data are presented as means (standard deviation, SD) or percentages except for a.

^a^As the distributions of the loss variances (LV) were skew, medians (interquartile range) of the LVs are presented in this row.

**Table 2:** The changes of global visual field indices with per unit increase of refractive error and optical axial length.

	OD		OS	
	Mean sensitivity	Mean defect	Mean sensitivity	Mean defect
Refractive error	0.06 (−0.18, 0.30)	−0.02 (−0.26, 0.21)	0.11 (−0.12, 0.34)	−0.09 (−0.32, 0.14)
Optical axial length	0.08 (−0.38, 0.53)	−0.13 (−0.58, 0.31)	−0.10 (−0.56, 0.35)	0.07 (−0.38, 0.52)

Data were presented as the changes of mean sensitivity (dB), mean defect (dB), and their 95% confidence intervals (in brackets) per unit increase of refractive error (diopter) and optical axial length (mm).

OD: oculus dexter (the right eye).

OS: oculus sinister (the left eye).

**Table 3:** The association between mean sensitivity (MS) and RNFL thickness.

Per 10 *μ*m increase in RNFL thickness			*n*	MS change (dB)	95% CIs	*R*	*R* ^2^	*P* value
General linear model	OD	Model I	57	−0.59	(−0.98, −0.20)	−0.37	0.13	0.005
		Model II		−0.59	(−0.98, −0.20)		0.23	0.008
	OS	Model I	57	−0.77	(−1.22, −0.32)	−0.40	0.16	0.002
		Model II		−0.78	(−1.26, −0.31)		0.26	0.002

Mixed model^*^		Model I	114	−0.39	(−0.70, −0.08)			0.014
		Model II		−0.39	(−0.70, −0.08)			0.012
		Model III		−0.39	(−0.71, −0.07)			0.019

^*^The associations were expressed by the change of MS (dB) per 10 *μ*m (approximately 1 SD) increase of RNFL thickness.

Model I: unadjusted model.

Model II: adjusted for age, sex, refraction (diopters), and area of optic disc (mm^2^).

Model III: adjusted for age, sex, and ocular magnification scale based on Bennett's formula.

^*^The mixed model combined the data of both right eyes and left eyes accounting for intraindividual binocular correlation.

SD: standard deviation; RNFL: retinal nerve fiber layer; CI: confidence interval; OD: oculus dexter (the right eye); OS: oculus sinister (the left eye).

**Table 4:** The association between mean defect (MD) and RNFL thickness.

Per 10 *μ*m increase in RNFL thickness			*n*	MD change (dB)	95% CIs	R	R^2^	P value
General linear model	OD	Model I	57	0.53	(0.14, 0.92)	0.34	0.11	0.011
		Model II		0.59	(0.18, 1.01)		0.22	0.007
	OS	Model I	57	0.74	(0.29, 1.19)	0.39	0.15	0.003
		Model II		0.79	(0.31, 1.26)		0.25	0.002

Mixed model^*^		Model I	114	0.37	(0.07, 0.67)			0.018
		Model II		0.42	(0.11, 0.74)			0.010
		Model III		0.40	(0.08, 0.72)			0.016

The associations were expressed by the change of MD (dB) per 10 *μ*m (approximately 1 SD) increase of RNFL thickness.

Model I: unadjusted model.

Model II: adjusted for age, sex, refraction (diopters), and area of optic disc (mm^2^).

Model III: adjusted for age, sex, and ocular magnification scale based on Bennett's formula.

^*^The mixed model combined the data of both right eyes and left eyes accounting for intraindividual binocular correlation.

SD: standard deviation; RNFL: retinal nerve fiber layer; CI: confidence interval; OD: oculus dexter (the right eye); OS: oculus sinister (the left eye).

## References

[1] He M., Zheng Y., Xiang F. (2009). Prevalence of myopia in urban and rural children in mainland china. *Optometry and Vision Science*.

[2] Cheng D., Schmid K. L., Woo G. C. (2007). Myopia prevalence in Chinese-Canadian children in an optometric practice. *Optometry and Vision Science*.

[3] Lam C. S., Goldschmidt E., Edwards M. H. (2004). Prevalence of myopia in local and international schools in Hong Kong. *Optometry & Vision Science*.

[4] Fan D. S., Lam D. S., Lam R. F., Lau J. T., Chong K. S., Cheung E. Y., Lai R. Y., Chew S.-J. (2004). Prevalence, incidence, and progression of myopia of school children in Hong Kong. *Investigative Ophthalmology and Visual Science*.

[5] Wu H.-M., Seet B., Yap E. P.-H., Saw S.-M., Lim T.-H., Chia K.-S. (2001). Does education explain ethnic differences in myopia prevalence? A population-based study of young adult males in Singapore. *Optometry and Vision Science*.

[6] Detry-Morel M. (2011). Is myopia a risk factor for glaucoma?. *Journal Francais d'Ophtalmologie*.

[7] Chang R. T. (2011). Myopia and glaucoma. *International Ophthalmology Clinics*.

[8] Marcus M. W., de Vries M. M., Junoy Montolio F. G., Jansonius N. M. (2011). Myopia as a risk factor for open-angle glaucoma: a systematic review and meta-analysis. *Ophthalmology*.

[9] Ohno-Matsui K., Shimada N., Yasuzumi K., Hayashi K., Yoshida T., Kojima A., Moriyama M., Tokoro T. (2011). Long-term development of significant visual field defects in highly myopic eyes. *The American Journal of Ophthalmology*.

[10] Kim M. J., Lee E. J., Kim T.-W. (2010). Peripapillary retinal nerve fibre layer thickness profile in subjects with myopia measured using the Stratus optical coherence tomography. *British Journal of Ophthalmology*.

[11] Kang S. H., Hong S. W., Im S. K., Lee S. H., Ahn M. D. (2010). Effect of myopia on the thickness of the retinal nerve fiber layer measured by cirrus HD optical coherence tomography. *Investigative Ophthalmology & Visual Science*.

[12] Rauscher F. M., Sekhon N., Feuer W. J., Budenz D. L. (2009). Myopia affects retinal nerve fiber layer measurements as determined by optical coherence tomography. *Journal of Glaucoma*.

[13] Leung C. K.-S., Mohamed S., Leung K. S., Cheung C. Y.-L., Chan S. L.-W., Cheng D. K.-Y., Lee A. K.-C., Leung G. Y.-O., Rao S. K., Lam D. S. C. (2006). Retinal nerve fiber layer measurements in myopia: an optical coherence tomography study. *Investigative Ophthalmology and Visual Science*.

[14] Choi S.-W., Lee S.-J. (2006). Thickness changes in the fovea and peripapillary retinal nerve fiber layer depend on the degree of myopia. *Korean Journal of Ophthalmology: KJO*.

[15] Wang G., Qiu K. L., Lu X. H., Sun L. X., Liao X. J., Chen H. L., Zhang M. Z. (2011). The effect of myopia on retinal nerve fibre layer measurement: a comparative study of spectral-domain optical coherence tomography and scanning laser polarimetry. *The British Journal of Ophthalmology*.

[16] Vetrugno M., Trabucco T., Sisto D., Troysi V., Sborgia G. (2007). The influence of low to moderate myopia on retinal nerve fiber layer as assessed by scanning laser polarimetry with variable corneal compensator. *Ophthalmologica*.

[17] Bennett A. G., Rudnicka A. R., Edgar D. F. (1994). Improvements on Littmann's method of determining the size of retinal features by fundus photography. *Graefe's Archive for Clinical and Experimental Ophthalmology*.

[18] Nowroozizadeh S., Cirineo N., Amini N., Knipping S., Chang T., Chou T., Caprioli J., Nouri-Mahdavi K. (2014). Influence of correction of ocular magnification on spectral-domain OCT retinal nerve fiber layer measurement variability and performance. *Investigative Ophthalmology & Visual Science*.

[19] Taliantzis S., Papaconstantinou D., Koutsandrea C., Moschos M., Apostolopoulos M., Georgopoulos G. (2009). Comparative studies of RNFL thickness measured by OCT with global index of visual fields in patients with ocular hypertension and early open angle glaucoma. *Clinical Ophthalmology*.

[20] Martin-Boglind L. (1991). High-pass resolution perimetry in uncomplicated myopia. *Acta Ophthalmologica*.

[21] Huang S.-J. (1993). Early change of visual function in high myopia—measured and analyzed by octopus automated perimeter. *Journal of Japanese Ophthalmological Society*.

[22] Araie M., Arai M., Koseki N., Suzuki Y. (1995). Influence of myopic refraction on visual field defects in normal tension and primary open angle glaucoma. *Japanese Journal of Ophthalmology*.

[23] Rudnicka A. R., Edgar D. F. (1995). Automated static perimetry in myopes with peripapillary crescents—part I. *Ophthalmic and Physiological Optics*.

[24] Aung T., Foster P. J., Seah S. K., Chan S.-P., Lim W.-K., Wu H.-M., Lim A. T. H., Lee L., Chew S.-J. (2001). Automated static perimetry: the influence of myopia and its method of correction. *Ophthalmology*.

[25] Czepita D., Chmielewska I. (2004). Changes in the static visual field of patients with low and medium myopia. *Annales Academiae Medicae Stetinensis.*.

[26] Chao D. L., Shrivastava A., Kim D. H., Lin H., Singh K. (2010). Axial length does not correlate with degree of visual field loss in myopic chinese individuals with glaucomatous appearing optic nerves. *Journal of Glaucoma*.

[27] Ito A., Kawabata H., Fujimoto N., Adachi-Usami E. (2001). Effect of myopia on frequency-doubling perimetry. *Investigative Ophthalmology & Visual Science*.

[28] Koller G., Haas A., Zulauf M., Koerner F., Mojon D. (2001). Influence of refractive correction on peripheral visual field in static perimetry. *Graefe's Archive for Clinical and Experimental Ophthalmology*.

[29] Ajtony C., Balla Z., Somoskeoy S., Kovacs B. (2007). Relationship between visual field sensitivity and retinal nerve fiber layer thickness as measured by optical coherence tomography. *Investigative Ophthalmology and Visual Science*.

[30] Lopez-Peña M. J., Reras A., Larrosa J. M., Polo V., Pablo L. E. (2011). Relationship between standard automated perimetry and retinal nerve fiber layer parameters obtained with optical coherence tomography. *Journal of Glaucoma*.

[31] McKone E., Davies A. A., Fernando D. (2008). Blurry means good focus: myopia and visual attention. *Perception*.

[32] Phillips W. A., Chapman K. L. S., Daniel Berry P. (2004). Size perception is less context-sensitive in males. *Perception*.

[33] Ji L. J., Peng K., Nisbett R. E. (2000). Culture, control, and perception of relationships in the environment. *Journal of Personality and Social Psychology*.

[34] McKone E., Aimola Davies A., Fernando D., Aalders R., Leung H., Wickramariyaratne T., Platow M. J. (2010). Asia has the global advantage: race and visual attention. *Vision Research*.

